# First Reported Case of Dissociative Symptoms Associated With Zuranolone Use in Peripartum Depression

**DOI:** 10.7759/cureus.110076

**Published:** 2026-06-01

**Authors:** Harim I Ok, Yaswitha Mikkilineni, Priya Batheja, Najeeb Manalai, Allison Foroobar, Beth Yanoff, Suneeta Kumari, Partam Manalai

**Affiliations:** 1 Psychiatry, Liberty University College of Osteopathic Medicine, Fredericksburg, USA; 2 Psychiatry, Edward Via College of Osteopathic Medicine, Fredericksburg, USA; 3 Psychiatry, Intuitive Insight Inc., McLean, USA; 4 Psychiatry, Mary Washington Healthcare, Fredericksburg, USA; 5 Psychiatry, Hackensack Meridian Ocean Medical Center, Brick, USA

**Keywords:** brexanolone, central nervous system, dissociative disorder, dissociative symptoms, gaba receptors, neurosteroid, peripartum depression, postpartum depression, ptsd, zuranolone

## Abstract

Peripartum depression is commonly observed in women during or after pregnancy. While selective serotonin reuptake inhibitors remain a mainstay of treatment, novel neurosteroid-based therapies such as brexanolone and zuranolone offer promising, rapid-acting alternatives.

We have reported the first known case of dissociative symptoms temporally associated with zuranolone use. A woman in her early 50s with a history of post-traumatic stress disorder (PTSD) and bipolar 1 disorder presented to the emergency department on the seventh day of zuranolone therapy, exhibiting profound disorientation, impaired autobiographical recall, unsteady gait, and communication difficulties. She had restarted zuranolone after discontinuing it earlier due to initial side effects, including dizziness and mood worsening. Her laboratory and imaging workup were unremarkable, and her symptoms resolved within 10 hours of discontinuation of zuranolone with supportive care alone.

This case raises important concerns about the safety of neurosteroids in individuals with a history of trauma, particularly due to the potential overlap between GABAergic modulation and dissociative symptoms. Dysregulation of GABA_A_ receptors has been implicated in both PTSD and memory processes, suggesting a plausible neurobiological mechanism underlying dissociative effects of zuranolone. Further research is necessary to clarify the associations and determine the safety profile of neurosteroids in patients prone to dissociation.

Given the growing use of zuranolone in primary care and obstetrical and gynecological settings where psychiatric consultation may be limited or absent, clinicians should be vigilant for dissociative symptoms, especially in trauma-exposed populations. This report underscores the need for thorough psychiatric evaluation before initiating neurosteroid treatment and highlights gaps in access to psychiatric care that may hinder optimal management of complex peripartum presentations.

## Introduction

Depression commonly co-occurs with post-traumatic stress disorder (PTSD), including peripartum depression (PPD) [1]. A variety of treatment modalities exist for PPD, including neurosteroids such as brexanolone [2] and zuranolone [3]. These medications are believed to exert their effects primarily through positive allosteric modulation of the GABA_A_ receptors [4].

Medications acting on the gamma-aminobutyric acid (GABA) system have been associated with dissociative side effects, including dissociative amnesia. Dissociation has been well documented with agents such as zolpidem. Additionally, several anesthetic agents also mediate their effects via the GABA receptors [5]. Given that GABAergic dysregulation may play a role in the pathophysiology of PTSD, a condition associated with a predisposition to dissociative symptoms, this connection warrants further investigation when patients are treated with neurosteroids.

In this report, we present the first documented case of dissociation temporally associated with the use of zuranolone. We also briefly review treatment options for PPD, with particular focus on the role of neurosteroids. Written informed consent for publication was obtained from the patient in accordance with our institutional policies. This case report does not meet the federal definition of human subjects research and therefore did not require Institutional Review Board approval.

## Case presentation

A woman in her early 50s presented to the emergency department with acute dissociative symptoms, which included disorientation and impaired recall, gait, and communication. Her psychiatric history was significant for PTSD in the context of childhood trauma, generalized anxiety disorder, panic disorder, borderline personality disorder, and bipolar 1 disorder diagnosed in 2008 at age 25 years.

The patient was diagnosed with PTSD due to a childhood marked by severe psychological and physical abuse, including threats of institutionalization when she defied rigid family expectations. She was forced into an arranged marriage by her family in her early 20s and had two sons from that marriage. Although she had suspected early mood symptoms, she did not seek help until experiencing PPD at age 23 years following the birth of her first child. After her second delivery at age 25 years, she again developed PPD, for which she was started on fluoxetine, as she was initially diagnosed with depression. However, this precipitated a manic episode in 2008, characterized by hypersexuality, reckless behavior, pressured speech, grandiosity, indiscretions, insomnia, high irritability, and suicidal gestures. She was hospitalized and diagnosed with bipolar I disorder that same year. Since then, she has had approximately 10 psychiatric admissions, including 2 involuntary hospitalizations, and 2 suicide attempts -- the last at age 44 years.

Over the years, she has trialed multiple medications, including lithium, aripiprazole, quetiapine, and lamotrigine, with the latter being most effective.

The patient’s most recent episode was bipolar 1: severe depressive episode with peripartum onset. As mentioned before, she had been intermittently treated for bipolar disorder throughout adulthood and had a history of significant childhood trauma, leading to the development of PTSD. She engaged in psychotherapy for her psychiatric conditions episodically with good outcomes. Previously stabilized on appropriate doses of lamotrigine and sertraline, she discontinued treatment after losing access to her psychiatrist. She did not receive psychiatric care during pregnancy and, following delivery, faced prolonged wait times for outpatient services in her geographic area. Consequently, her gynecologist managed her mood symptoms to the best of their ability.

Following the birth of her third child, the patient continued to experience severe depressive symptoms. Without access to a psychiatrist or the ability to follow up with a therapist, she was initially prescribed sertraline by her gynecologist for two months, at six months postpartum, but her depressive symptoms did not improve. Subsequently, her gynecologist initiated treatment with zuranolone for severe postpartum depression.

After the initial dose of zuranolone, the patient reported worsening depression, dizziness, foggy memory, increased irritability, and dissociation. She discontinued the medication due to lack of improvement and the aforementioned side effects. However, she later chose to restart zuranolone a week later due to the severity of her postpartum depression, believing it might yield a different outcome than the initial dose. On the seventh day of treatment, she developed neuropsychiatric symptoms such as memory impairment, which were noticed by her husband, prompting her to seek emergency care. More specifically, she describes her memory of this period as very foggy and recalls her husband calling 911. She remembers only wanting to see her daughter, being in the ambulance, and urinating on herself. Her husband reported that she appeared disoriented and “like a zombie,” repeatedly saying, “help me.”

Dissociation and sedation are distinct clinical phenomena, though they can overlap in presentation. Dissociation refers to a disruption in the normal integration of consciousness, memory, and perception -- often manifesting as feelings of detachment or unreality while the individual remains awake. Sedation involves a pharmacologically induced reduction in alertness that can impair awareness, responsiveness, and memory. In our case, the patient initially described symptoms consistent with dissociation, including feeling “checked out,” detached, and as if she were floating, even before starting zuranolone, which aligns with derealization commonly associated with PTSD. However, after taking her second dose, she reported being so “out of it” that she could not recall what occurred, suggesting a shift toward profound sedation or altered consciousness rather than a purely dissociative state.

**Figure 1:**
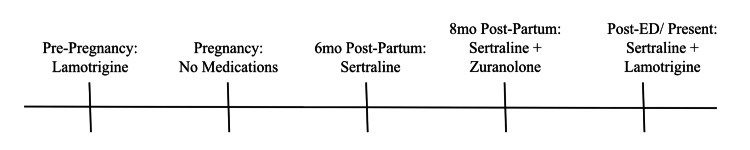
Timeline of psychiatric medications given to patient ED, emergency department.

Upon evaluation in the emergency room (ER), the patient exhibited marked disorientation, an inability to get out of bed, impaired recall of autobiographical information, difficulty communicating with family members, and an unsteady gait. She also endorsed symptoms, including sadness, feeling overwhelmed, anhedonia, low energy, low motivation, anxiety, and oversleeping the past few days before the ER visit. She did not report perceptual disturbances or suicidal ideation. Figure 1 illustrates the timeline of psychiatric medications administered to the patient.

All pertinent laboratory values, detailed below (Table 1), were unremarkable except for a mildly decreased mean corpuscular hemoglobin (27 picograms) and mean corpuscular hemoglobin concentration (30 grams per deciliter). These findings were consistent with her baseline values obtained three years before her admission (29, 24, respectively). Imaging studies, including a head CT scan, electrocardiogram, and chest radiograph, were all within normal limits. The laboratory evaluation included point-of-care glucose testing, complete blood count, comprehensive metabolic panel, prothrombin time with international normalized ratio, activated partial thromboplastin time, troponin, lipase, magnesium, lactic acid, human chorionic gonadotropin, ethanol level, urine chemistry, and urine drug screen.

**Table 1:** Patient laboratory values BUN, blood urea nitrogen; Cl, chlorine; CO2, carbon dioxide; Cr, creatinine; HCG, human chorionic gonadotropin; Hg, hemoglobin; K, potassium; MCH, mean corpuscular hemoglobin; MCHC, mean corpuscular hemoglobin concentration; Na, sodium; POCT, point-of-care testing; PT, prothrombin time; PTT, partial thromboplastin time; TCA, tricyclic antidepressant; WBC, white blood cell.

Lab	Patient values	Reference range
WBC	7.3	4-11 K/uL
Hg	11.2	11-15 g/dL
Platelets	244	130-400 K/uL
MCH	27	28-32 pg
MCHC	30	32-36 g/dL
POCT	78	60-110 mg/dL
Na	143	137-145 mmol/L
K	4.5	3.5-5.3 mmol/L
Cl	105	98-107 mmol/L
CO_2_	27	22-30 mmol/L
BUN	17	7-22 mg/dL
Cr	0.7	0.7-1.2 mg/dL
Glucose	75	65-105 mg/dL
PT	10.9	9.5-11.5 seconds
PTT	23.9	23.0-33.0 seconds
Troponin I	<0.012	0.000-0.033 ng/mL
Lipase	114	23-300 U/L
Magnesium	2	1.6-2.3 mg/dL
Lactate	1	0.70-2.10 mmol/L
HCG qualitative serum	Negative	Negative
Ethanol lvl	Negative	Negative
Urine chemistries (leukocyte esterase, nitrite, protein, glucose, ketones, bilirubin, and blood)	Negative	Negative
Rapid drug screen (cannabinoid, phencyclidine, cocaine, methamphetamine, opiate, amphetamine, benzodiazepines, TCA, methadone, barbiturate, oxycodone, and buprenorphine)	Negative	Negative

The patient did not require admission to a medical unit from the emergency department, as her neuropsychiatric symptoms gradually improved over 10 hours with discontinuation of zuranolone and supportive care. Given the severity of her depressive symptoms, voluntary inpatient psychiatric hospitalization was clinically indicated. However, this was not acceptable to the patient due to the lack of available childcare for her infant. Admission to our partial hospitalization program was offered as an alternative, but she declined for the same reason. Her significant other identified an out-of-state program that would allow admission with her child for a month-long stay. In the meantime, the patient was advised to enroll in our outpatient psychiatric program, discontinue zuranolone, resume lamotrigine, albeit at a lower dose, and increase the dose gradually in consultation with her psychiatrist. The patient was already on sertraline before hospital admission and was advised to continue it, as she had previously responded well to the combination of sertraline and lamotrigine. Although second-generation antipsychotics were discussed as a potential treatment option for severe depressive symptoms, the patient declined to initiate them.

The patient was also counseled on the benefits of psychotherapy and encouraged to re-engage and establish a therapeutic relationship with a therapist, given the strong evidence supporting the combined efficacy of psychotherapy and pharmacotherapy in the management of mood disorders. The patient was discharged from the ER in medically stable condition, accompanied by her family.

Considering the ER visit occurred on the weekend, the patient requested a callback on the following Monday. Though initial outreach attempts were unsuccessful, follow-up was successfully made a few months later.

At that time, the patient reported that she did not attend a residential program but now follows with an independent provider. She is continuing on sertraline 50 mg once daily and lamotrigine 200 mg once daily, and the combination is working well for her with no side effects reported. It should be noted that the patient does not appear to have been screened for the dissociative subtype of PTSD. Due to financial limitations, she has not been following up with therapy at this time but has identified coping mechanisms that have been helping her. Since discontinuing zuranolone, she reports no symptoms similar to those she experienced on the medication and reports doing well overall. The patient has given and confirmed consent for publication.

## Discussion

Major depressive episode, with peripartum onset (PPD), is defined in the Diagnostic and Statistical Manual of Mental Disorders, Fifth Edition, Text Revision (DSM-5-TR) as a major depressive episode occurring during pregnancy or within four weeks postpartum [6]. It affects approximately 26% of pregnant women [1]. This period presents unique challenges, as women must balance their own mental health needs with treatment options that minimize risk to the fetus or newborn. Although pregnant women may require higher doses of antidepressants due to physiological changes, concerns about fetal exposure often lead to subtherapeutic prescribing in cases of PPD. Additionally, many women, including our patient, prefer non-pharmacological interventions during pregnancy [7].

Despite the high prevalence of PPD, there is limited high-quality evidence supporting specific treatment modalities, particularly regarding pharmacologic recommendations. Although comparative data between classes of antidepressants are lacking, selective serotonin reuptake inhibitors (SSRIs) appear to be more effective in achieving symptom remission or reduction and are considered fairly safe in pregnancy [8-10]. Fluoxetine, especially when combined with cognitive behavioral therapy (CBT), has shown greater improvements in symptom reduction than either modality alone [11]. Other non-pharmacological modalities, such as group therapy, interpersonal therapy, transactional analysis, psychodynamic therapy, psychoeducation, transcranial magnetic stimulation, and pharmacotherapy, are available as options as well.

One meta-analysis reported an overall effect size of 0.67 (p<0.001) for CBT with medication compared with medication alone, which indicates moderate-to-strong efficacy [8]. A randomized controlled trial investigating paroxetine demonstrated both clinically and statistically significant benefits in the treatment of PPD [12]. In smaller patient cohorts, sertraline was found to reduce the risk of developing PPD, whereas amitriptyline did not show a preventive effect. Repetitive transcranial magnetic stimulation has also shown a significant therapeutic effect on PPD [13,14]. Although mood stabilizers and second-generation antipsychotics can be beneficial in managing bipolar disorder, antidepressant monotherapy is not recommended due to rapid cycling and frequent mood swings [15].

Neuroactive steroids are a class of steroids capable of modulating neuronal activity. The brain is considered a steroidogenic organ, producing neurosteroids that influence both GABA_A_ and N-methyl-D-aspartate receptor expression and function, thereby regulating inhibitory and excitatory signaling in the CNS [16]. These compounds are produced locally within the CNS by both neurons and glial cells, which have higher concentrations in brain tissue than in serum, suggesting local regulation [17]. Multiple brain cell types, including neurons, astrocytes, oligodendrocytes, and microglia, synthesize various neurosteroids such as pregnenolone, dehydroepiandrosterone, androstenedione, and estrogen, at various levels, further highlighting the complex and cell-specific steroidogenic capacity of the CNS [17]. Among the neurosteroids, 17β-estradiol plays an anti-inflammatory role and initiates various signaling pathways, including calcium (Ca²⁺), cyclic adenosine monophosphate, and extracellular signal-regulated kinase signaling. Progesterone and allopregnanolone, derived from both CNS and peripheral sources, have been shown to downregulate inflammasome activation for the regulation of the inflammatory process [18].

There are only two novel medications, brexanolone and zuranolone (Table 2), specifically approved for the treatment of PPD that have shown highly favorable outcomes for pregnant women and new mothers with a rapid onset of action [2,3,19,20]. Both of these medications act through the CNS's neurosteroid system, with their effects potentially mediated via the GABA_A_ receptor.

**Table 2:** Pharmacological comparison of brexanolone and zuranolone Data source: [21] eGFR, estimated glomerular filtration rate; ESRD, end-stage renal disease; PPD, peripartum depression.

	Brexanolone	Zuranolone
Indication	PPD in patients 15 years or older	PPD in adults
Adverse effect	Somnolence, xerostomia, loss of consciousness, and flushing	Somnolence, dizziness, diarrhea, fatigue, nasopharyngitis, and urinary tract infection
Dosing administration	Administered at a healthcare facility as a single 60-hour continuous IV infusion	Taken orally once daily in the evening with a high-fat meal for 14 days
Dosing adjustments	Avoid use in patients with ESRD with an eGFR below 15 mL/min/1.73 m²	Reduce the dosage in patients with hepatic or renal impairment (eGFR <60 mL/min/1.73 m^2^). Avoid concurrent use of strong CYP3A4 inhibitors, CNS adverse effects, or concurrent use with other CNS depressants
Boxed warnings	Available exclusively through the Zulresso REMS program due to the risk of excessive sedation or loss of consciousness, which can lead to serious harm	May cause driving impairment due to CNS depressant effects. Patients should avoid driving or other hazardous activities for at least 12 hours after administration. Caution for increased risk of falls or dizziness
Monitoring criteria	Monitor every two hours during the infusion for signs of CNS depression, as well as for suicidal thoughts or behaviors	Monitor for CNS depressant effects and suicidal thoughts and behavior
Safety considerations	May cause fetal harm in pregnancy. Avoid using in patients with ESRD	May cause fetal harm: Patients should use effective contraception during treatment and for one week after the last dose

Allopregnanolone (brexanolone), a progesterone-derived neurosteroid that modulates GABA_A_ receptor activity, plays a key role in the pathophysiology of PPD. It binds to GABA_A_ receptors on microglia and astrocytes, mediating anti-inflammatory effects. Although its levels alone do not predict symptoms, the therapeutic success of brexanolone supports the role of allopregnanolone in the pathophysiology of PPD [2,4,19,20]. Brexanolone is minimally excreted in breast milk, making it an ideal option for new mothers, as they do not need to withhold breastfeeding during treatment [4,20]. Brexanolone is the first US Food and Drug Administration-approved neurosteroid for moderate-to-severe postpartum depression. It can produce a rapid reduction in PPD symptoms with relatively few side effects. However, due to the risk of severe sedation and loss of consciousness, it is administered in an inpatient setting via IV infusion over a 60-hour period [4,20]. A significant barrier to the use of brexanolone is its significantly higher cost, which is often not covered by insurance plans. The cost of brexanolone is approximately $38,000, compared with $4-$30 per month for an affordable SSRI. Nonetheless, the total benefits to patients from brexanolone may justify the upfront cost [22].

Zuranolone is a synthetic neurosteroid that acts as a positive allosteric modulator of GABA_A_ receptors and also enhances their surface expression through metabotropic mechanisms. These actions differ from medications such as benzodiazepines (BDZs), which only modulate GABA_A_ receptor activity at the GABA-binding site [4]. GABA_A_ receptors are heteropentameric structures composed of various combinations of subunits that include six alpha, three beta, and three gamma subunits. Zuranolone enhances the potency and efficacy of GABA at the GABA_A_ receptor [4]. It is thought to bind to the α subunit of the GABA_A_ receptor, whereas BDZs bind to both the α and γ subunits. When administered with diazepam, zuranolone can enhance the BDZ's effects synergistically. However, unlike BDZs, the effects of zuranolone are not dependent on the γ subunit [4]. Like brexanolone, zuranolone is a rapid-acting treatment, often producing noticeable effects within a week. However, similar challenges, particularly high cost and limited insurance coverage, make zuranolone less practical for widespread use. Although the total societal benefits may justify the upfront expense, a 14-day course of zuranolone costs approximately $16,000. The price tags of both brexanolone and zuranolone render these treatment options inaccessible to most patients [23].

The precise binding site of neurosteroids, including brexanolone and zuranolone, at the GABA_A_ receptor has not been fully elucidated; multiple sites on the α and β subunits have been implicated. Various agents that act on GABA_A_ receptors, including propofol, etomidate, BDZs, Z-drugs, and alcohol, have been associated with dissociative or altered states of consciousness. Since neurosteroids are also believed to exert their effects through GABA_A_ receptors, it is theoretically plausible that synthetic neurosteroids such as zuranolone and allopregnanolone may also induce dissociative states, particularly in those prone to dissociation, such as PTSD [24]. Neurosteroids not only modulate inhibitory GABAergic transmission but also influence excitatory glutamatergic systems and neuroinflammation, further impacting neural circuits implicated in mood, cognition, and consciousness.

In this context, the development of severe dissociative symptoms in our patient with a history of PTSD following zuranolone administration suggests that dissociation may represent a potential, albeit rare, neuropsychiatric side effect of this novel therapy. Nonetheless, there are factors that pose limitations to the case. Obtaining a detailed history from the patient was challenging due to her cognitive changes, which is a limitation in this case. The dissociative symptoms that she experienced may coincide with symptoms of sedation, confusional state, and hallucination, which are also side effects of sertraline [25]. Since the patient was on sertraline before the immediate initiation of zuranolone, her symptoms from zuranolone may have blended with the symptoms from sertraline. In addition, the washout period for sertraline is about one to two weeks. However, the symptoms of dissociation and PTSD were brought to the surface rapidly after initiation of zuranolone. This suggests that zuranolone may have had a great impact in triggering the dissociative and PTSD symptoms. Although this is a single case report, the pharmacological profile of zuranolone and the vulnerability of specific patient populations warrant careful monitoring for dissociative phenomena, particularly in those with pre-existing trauma-related disorders. Such monitoring can be challenging when psychiatrists are not available. Psychiatrists are more likely to recognize subtle early neuropsychiatric side effects of medications; however, due to the ongoing shortage of psychiatrists in the United States, primary care providers and obstetrical and gynecological (OB/GYN) practices are often tasked with delivering psychiatric care.

## Conclusions

To the best of the authors’ knowledge, this is the first case report noting a temporal relationship between zuranolone therapy for PPD and dissociative symptoms. Although our patient had a history of PTSD, the temporal association between the dissociative state and zuranolone treatment warrants further investigation. At face value, the mechanism of action of this medication provides a biochemical basis that could explain the association between zuranolone use and dissociative symptoms. 

In our case report, the patient’s gynecologist approached the patient’s care sensibly and appropriately; nonetheless, a psychiatric consultation could potentially have improved the outcome. For example, the patient’s history of trauma might have been addressed early during treatment for depression. Furthermore, given the patient’s history of bipolar disorder, the use of antidepressants warrants caution. The lack of access to psychiatric care, in the context of a psychiatrist shortage in the area, required our OB/GYN colleagues to manage the psychiatric needs of our patient. The current case report exemplifies the unmet need of our communities, where a person who may be persuaded to seek psychiatric care and engage in psychotherapy is not able to secure a follow-up. Such delays in care may result in frustration, and patients may forgo seeking help. A presentation like that of our patient highlights the need to allocate more resources toward expanding psychiatry residency training programs in the United States, as well as enhancing collaborative care among psychiatrists, primary care providers, and OB/GYN colleagues. With a more integrated approach, it is possible that our patient would have received psychotherapy and more targeted pharmacological treatment tailored to her specific needs.
